# Endogenous retroviruses in primates

**DOI:** 10.1186/1742-4690-8-S2-P7

**Published:** 2011-10-03

**Authors:** Katherine Brown, Ed Louis, Richard D Emes, Rachael Tarlinton

**Affiliations:** 1 School of Veterinary Medicine and Science, University of Nottingham, Sutton Bonington Campus, Loughborough, LE1 2 5RD, UK; 2 School of Biology University of Nottingham, University Park Campus, Nottingham, NG7 2RD, UK

## Background

Endogenous retroviruses (ERVs) have much lower mutation rates than exogenous retroviruses (XRVs), therefore they are an important tool in analysing the long-term evolutionary history of retroviruses. Their transmission patterns also act as useful markers when studying host phylogenetics.

Recently, the first examples of endogenous lentivirus were characterised. The earliest was rabbit endogenous lentivirus type K in the European rabbit *[Oryctolagus cuniculus*) [*1*]. Later, examples in the European brown hare (*Lepus europeaus*) [*2*] and in two species of prosimian primate, the grey mouse lemur(*Microcebus murinus*) [*3*] and fat-tailed dwarf lemur (*Cheirogaelus medius*) [*4*] were discovered. Other ERVs are much more widespread, particularly *gamma-* and *beta-* retroviruses, which are present in many vertebrate species The aim of this project is to look for and characterise further examples of primate ERVs and therefore gain insight into the evolutionary history of retroviruses and their primate hosts. This will involve data mining of primate genomes for previously unknown ERVs as well as PCR based screening of genera for which genome sequences are not available. Preliminary work suggests that prosimian primate genomes contain previously unclassified endogenous lentiviruses, so PCR-based screening will be used with DNA samples from lemurs, bushbabies and lorises to sequence and characterise these viruses.

## Materials and methods

The bioinformatics analysis component of this project used Exonerate [5] to perform pairwise sequence comparison between host chromosomes and query retroviral amino acid sequences. Sequences from the polymerase (*pol*), group-specific antigen (*gag*) and envelope (*env*) genes of representative XRVs from all known retroviral genera were used to identify regions of the host genome which are highly similar to retroviral genes. We have validated this method by successfully searching the human genome for known ERV insertions. Regions of interest are then extracted and analysed in detail.

The initial laboratory work used primers which act on a conserved region of the lentiviral *pol* gene (primers from [4]) on samples from 17 species of prosimian primate, to look for examples of lentiviral insertions. These were then sequenced and incorporated into the lentiviral phylogeny.

## Results

Bioinformatics analysis of the low-coverage *M. murinus* genome sequence identified 3542 ERV-like regions. This included 256 regions of less than 10,000 base pairs which incorporated *gag*, *pol* and env-like sequences in the correct orientation. The subset of *pol* genes which were within these 256 proviruses appear to be closely related to known *gamma-* and *beta-*retroviruses from other species.

Preliminary laboratory work has shown previously uncharacterised lentiviral insertions in several species of lemur - *Varecia variegata*, *Eulemur rufus* and *Mirza coquereli;* the loris *Perodictus potto* and the bushbaby *Galago moholi*, although these insertions are yet to be further characterised.

## Conclusions

Multiple potential ERV sequences have been found in the M. *murinus* genome. Preliminary laboratory results suggest that there are undescribed endogenous lentiviruses in further prosimian primate species. Methodology has been established which can be used to screen further species for proviral insertions and to explore these in detail.
